# Genome Sequences of Five Arboviruses in Field-Captured Mosquitoes in a Unique Rural Environment of South Korea

**DOI:** 10.1128/genomeA.01644-15

**Published:** 2016-01-28

**Authors:** Jun Hang, Terry A. Klein, Heung-Chul Kim, Yu Yang, Dereje D. Jima, Jason H. Richardson, Richard G. Jarman

**Affiliations:** a Walter Reed Army Institute of Research, Silver Spring, Maryland, USA; b U.S. Army Public Health District-Korea (P), 65th Medical Brigade, Unit 15281, APO AP 96205-5281, USA; c U.S. Army 5th Medical Detachment, 168th Multifunctional Medical Battalion, 65th Medical Brigade, Unit 25247, APO AP 96205-5247, USA; d Armed Forces Pest Management Board, Silver Spring, Maryland, USA

## Abstract

Here, we present the genome sequences of one mesonivirus and four novel arboviruses observed in *Culex bitaeniorhynchus* and *Culex pipiens*, captured in and near the demilitarized zone, Republic of Korea. Results suggest the ubiquitous presence of mesoniviruses and the discovery of a potentially new species of arboviruses in field-captured mosquitoes.

## GENOME ANNOUNCEMENT

Arthropod-borne viruses (arboviruses) infect a wide range of animal and plant hosts (1). Active vector and pathogen surveillance can provide early warning of public health threats (2–4). Viral identification from vectors can be achieved by various techniques, including viral culture, microarrays, and PCR assays. We utilized random amplification and next-generation DNA sequencing to identify known and novel arboviruses in mosquitoes.

Mosquitoes were trapped using Mosquito Magnets (Woodstream Corp., Lititz, PA, USA) in 2012 at four collection sites in and near the demilitarized zone, Republic of Korea. Mosquito species were separated and pooled by site and date of collection. Mosquitoes were triturated by bead-beating and centrifuged to obtain supernatants for RNA purification by nucleases pretreatment, followed by extraction using the QIAamp viral RNA kit (Qiagen Sciences, Germantown, MD, USA) (5). Nucleic acid extracts were subjected to random reverse transcription and random PCR amplification. Random amplicons were sequenced using the MiSeq sequencer with the Nextera XT DNA library prep and sequencing kit version 3 (Illumina, San Diego, CA, USA) (6). A bioinformatics pipeline comprising data processing, host sequence mapping, *de novo* assembly, and sequence blasting was used for sequence-based identification and taxonomical determination. The tools used for analysis included Ray *de novo* genome assembler version 2.2 (7), Roche GS analysis software version 2.9 (Roche 454 Life Sciences, Branford, CT, USA), BLAST programs (http://blast.ncbi.nlm.nih.gov/Blast.cgi), and Geneious version 8.1.7 (Biomatters, Auckland, New Zealand).

Five viral genome sequences containing complete protein-coding sequences (CDSs) were assembled with 0.77 to 1.06 million reads with average alignment coverage ranging from 582- to 8,086-fold. The assembled genome sequence for the virus identified in *Culex pipiens*, designed as Alphamesonivirus 1/A12.2520/ROK/2012 (family *Mesoniviridae*, genus *Alphamesonivirus*), has 20,090 bases and shares 98.96% nucleotide identity with Houston virus (KC807175.1), which suggests a worldwide distribution of mesoniviruses in multiple species of mosquitoes (8). The genome sequence for the virus in *Culex bitaeniorhynchus*, designated Imjin River virus 1/A12.2496/ROK/2012 has 11,877 bases with only 73.24% nucleotide identity in the most conserved RNA-dependent RNA polymerase (RdRp) gene of the 12,004-bp genome of Wuhan mosquito virus 8 (KM817610.1), an unclassified ssRNA negative-strand virus in the proposed *Chuviridae* family (9). The genome sequence for the virus in *Culex bitaeniorhynchus*, designated Tongilchon virus 1/A12.2676/ROK/2012, has 11,355 bases and contains all four complete CDS sequences that have 65.8 to 74.8% nucleotide identity with RdRp, glycoprotein (G), phosphoprotein (P), and nucleoprotein (N) genes (KF360970 to KF360973) of North Creek virus (family *Rhabdoviridae*).

Moreover, two novel small viruses were found in *Culex pipiens*, designated Daeseongdong virus 1/A12.2708/ROK/2012 and Daeseongdong virus 2/A12.2549/ROK/2012. The genome sequences are 9,632 bases with three complete CDSs and 4,742 bases with two complete CDSs, respectively. A BLASTp search of the putative proteins against the GenBank non-redundant (nr) protein sequences database suggest that they are distantly related to *Negevirus*, a proposed new taxon (10, 11), and Drosophila A virus (12), an unclassified ssRNA positive-strand virus, respectively. It is intriguing to investigate the host specificity, role in viral evolution, and potential pathogenicity of these arboviruses from the rural area of South Korea.

### Nucleotide sequence accession numbers.

The five genome sequences were deposited in GenBank under accession numbers KU095838 to KU095842.
